# Supplementary material for: Modulatory Effects of the Kuwanon-Rich Fraction from Mulberry Root Bark on the Renin–Angiotensin System
Source: Foods. 2024 May 16;13(10):1547. doi: 10.3390/foods13101547 (PMC11121332; doi:10.3390/foods13101547)
Supplement: Supplementary file 1 [file foods-13-01547-s001.zip › foods-2991719-supplementary.pdf]

foods MDPI

Supplementary tables

Table S1. CI (95%) analysis of each data set

Figure No.	Experiment		Group	Mean (95% CI)
Figure 1	A. Total polyphenol	MeOH	Root_Cheongil	90.58 (89.39-91.77)
			Root_Cheongol	86.42 (80.00-92.83)
			Root_Gwasang2	91.38 (88.86-93.91)
			Root_Daeshim	94.18 (93.77-94.58)
			Twig_Cheongil	44.90 (43.71-46.10)
			Twig_Cheongol	52.23 (50.36-54.11)
		CH2Cl2	Root_Cheongil	86.89 (84.69-89.09)
			Root_Cheongol	89.96 (83.97-95.95)
			Root_Gwasang2	89.12 (86.09-92.15)
			Root_Daeshim	91.28 (87.11-95.46)
			Twig_Cheongil	67.59 (64.58-70.59)
			Twig_Cheongol	62.60 (58.25-66.94)
		EtOAc	Root_Cheongil	110.00 (106.04-113.97)
			Root_Cheongol	105.11 (93.82-116.40)
			Root_Gwasang2	103.21 (99.25-107.18)
			Root_Daeshim	105.12 (103.67-106.57)
			Twig_Cheongil	92.57 (87.23-97.91)
			Twig_Cheongol	90.37 (85.79-94.95)
		BuOH	Root_Cheongil	85.32 (80.83-89.82)
			Root_Cheongol	75.68 (69.97-81.40)
			Root_Gwasang2	84.53 (77.10-85.95)
			Root_Daeshim	80.20 (77.30-83.10)
			Twig_Cheongil	67.24 (62.91-71.57)
			Twig_Cheongol	73.48 (67.40-79.56)
		Water	Root_Cheongil	64.48 (61.74-67.22)
			Root_Cheongol	52.46 (47.97-56.96)

		Root_Gwasang2	74.66 (74.11-78.21)
		Root_Daeshim	39.18 (38.27-40.09)
		Twig_Cheongil	30.50 (29.51-31.48)
		Twig_Cheongol	35.17 (32.86-37.48)
B. Flavonoid	MeOH	Root_Cheongil	120.30 (109.54-131.05)
		Root_Cheongol	148.46 (139.63-157.30)
		Root_Gwasang2	154.57 (147.72-161.42)
		Root_Daeshim	263.56 (256.07-271.05)
		Twig_Cheongil	18.38 (18.16-18.55)
		Twig_Cheongol	23.20 (21.94-24.47)
	CH2Cl2	Root_Cheongil	354.65 (343.64-365.67)
		Root_Cheongol	406.55 (385.95-427.16)
		Root_Gwasang2	442.03 (439.49-444.57)
		Root_Daeshim	402.72 (396.23-409.21)
		Twig_Cheongil	55.48 (50.88-60.07)
		Twig_Cheongol	45.20 (41.32-49.07)
	EtOAc	Root_Cheongil	366.44 (360.12-372.76)
		Root_Cheongol	427.93 (405.48-450.37)
		Root_Gwasang2	376.39 (335.54-417.24)
		Root_Daeshim	471.23 (465.24-477.23)
		Twig_Cheongil	127.74 (124.74-130.74)
		Twig_Cheongol	132.76 (121.39-144.14)
	BuOH	Root_Cheongil	57.93 (56.69-59.17)
		Root_Cheongol	41.27 (39.63-42.91)
		Root_Gwasang2	36.48 (35.73-37.23)
		Root_Daeshim	53.42 (50.73-56.10)
		Twig_Cheongil	20.06 (19.48-20.64)
		Twig_Cheongol	25.40 (23.82-26.98)
	Water	Root_Cheongil	18.10 (17.50-18.69)
		Root_Cheongol	13.14 (12.69-13.60)
		Root_Gwasang2	24.21 (23.62-24.81)

			Root_Daeshim	12.27 (11.22-13.31)
			Twig_Cheongil	9.38(8.99-9.77)
			Twig_Cheongol	13.55 (12.29-14.82)
Figure 2	A. ACE relative inhibition		BC	100 (84.15-115.85)
			IC	71. (43.45-98.78)
		MeOH	100 µg/mL	96.02 (90.22-101.82)
			50 µg/mL	94.86 (87.74-101.97)
			10 µg/mL	23.39 (13.73-33.06)
		CH2Cl2	100 µg/mL	100.89 (99.69-102.09)
			50 µg/mL	86.12(78.17-94.07)
			10 µg/mL	81.32(76.60-86.04)
		EtOAc	100 µg/mL	97.72 (95.87-99.57)
			50 µg/mL	102.05 (95.70-108.40)
			10 µg/mL	95.01 (92.37-97.65)
		BuOH	100 µg/mL	100.89 (95.09-106.69)
			50 µg/mL	94.78 (92.78-96.78)
			10 µg/mL	6.38 (-1.54-14.30)
		DW	100 µg/mL	9.37 (7.26-11.49)
			50 µg/mL	NA
			10 g/mL	NA
	B. ACE relative inhibition		BC	100 (92.65-107.35)
			IC	81.53 (76.46-86.60)
			Twig_Cheongol	42.62 (40.70-44.54)
			Twig_Cheongil	63.98 (59.83-68.13)
			Root_Cheongol	102.42 (91.08-113.75)
			Root_Cheongil	100.03 (98.76-101.31)
			Root_Daeshim	98.33 (91.12-105.54)
			Root_Gwasang2	92.21 (87.08-97.34)
Figure 3	A. ACE relative inhibition		BC	100 (97.24-102.76)
			IC	63.78 (55.51-72.06)

		Resveratrol		NA
		Oxyresveratrol		NA
		Mulberroside A		NA
		Kuwanon G		7.85 (-8.01-23.71)
		Kuwanon H		19.39 (15.93-22.84)
	B. ACE relative inhibition	BC		100 (70.84-129.16)
		IC		83.48 (79.11-87.96)
		Kuwanon G	10 μg/mL	4.22 (2.32-6.13)
			100 μg/mL	39.24 (34.75-43.73)
		Kuwanon H	10 μg/mL	20.89 (17.65-24.12)
			100 μg/mL	86.63 (80.32-92.94)
Figure 5	Weight gain	HSD		6.05 (5.23-6.88)
		RBE		5.27 (4.67-5.89)
		RBF		5.44 (4.58-6.31)
	Liver weight	HSD		0.76 (0.69-0.84)
		RBE		0.77 (0.73-0.82)
		RBF		0.76 (0.71-0.82)
	Kidney weight	HSD		0.28 (0.26-0.29)
		RBE		0.27 (0.25-0.28)
		RBF		0.27 (0.25-0.29)
	Heart weight	HSD		0.11 (0.10-0.11)
		RBE		0.10 (0.10-0.11)
		RBF		0.09 (0.09-0.11)
	Serum Renin	HSD		5.96 (4.74-7.19)
		RBE		4.53 (3.47-5.59)
		RBF		3.95(3.31-4.59)
	Serum AGT	HSD		9.87 (8.83-10.92)
		RBE		8.43 (7.06-9.81)
		RBF		7.37 (5.91-8.82)
